# A review on waste valorization, biotechnological utilization, and management of potato

**DOI:** 10.1002/fsn3.3546

**Published:** 2023-07-14

**Authors:** Anamika Chauhan, Fakhar Islam, Ali Imran, Ali Ikram, Tahir Zahoor, Sadaf Khurshid, Mohd Asif Shah

**Affiliations:** ^1^ Department of Home Science ChamanLal Mahavidyalay Landhora Haridwar India; ^2^ Sri Dev Suman University Tehri India; ^3^ Department of Food Science Government College University Faisalabad Pakistan; ^4^ Department of Clinical Nutrition NUR International University Lahore Pakistan; ^5^ University Institute of Food Science and Technology, The University of Lahore Lahore Pakistan; ^6^ Department of Home Economics Government College University Faisalabad Pakistan; ^7^ Department of Economics Kabridahar University Jigjiga Ethiopia; ^8^ School of Business Woxsen University Hyderabad Telangana India; ^9^ Division of Research and Development Lovely Professional University Phagwara India; ^10^ School of Engineering and Technology Sharda University Greater Noida India

**Keywords:** biotechnological potato waste management, eco‐friendly biotechnological waste management, potato waste management, potato wastes

## Abstract

One of the most popular, cost‐effective crops that are consumed globally is the potato. Due to the expanding food crisis, there is an increase in the demand for potato‐based agro‐food items. At the same time, it is noted that this pathway of ecological pollution from large‐scale wastes is challenging to manage. The food sector generates a lot of waste, which can be controlled better via biotechnological methods. The potato industry is one of the industries that generate a large amount of garbage that is harmful to the environment. Several by‐products of industrial potato production, such as potato peels (PPs), starch, flakes, and granules, are disposed of despite being rich sources of nutrients and bioactive ingredients. These wastes can subsequently be used in biotechnological processing to produce microbial polysaccharides, yeast cellular biomass, lipids, protein, enzymes, organic acids, and carotenoids as components of the microbial medium. Similarly, food processing based on potatoes uses a lot of water, which is an issue because it pollutes wastewater. The most popular method for reducing trash that is both affordable and environmentally beneficial at the moment is biotechnology. The purpose of this review study is to illustrate the potential of applying biotechnological techniques to tackle the potato waste problem while simultaneously enhancing the economy. By discussing recent breakthroughs as well as current flaws in this method of controlling potato trash, this paper seeks to give a future vision of the justifiable use of biotechnological‐based potato waste management and utilization strategies.

## Graphical abstract

One of the most popular, cost‐effective agricultural crops that are consumed globally is the potato. Due to the expanding food crisis, there is an increase in the demand for potato‐based agro‐food items. At the same time, it is noted that this pathway of ecological pollution from large‐scale wastes is challenging to manage. Several by‐products of industrial potato production, such as PPs, starch, flakes, and granules, are disposed of despite being rich sources of nutrients and bioactive ingredients.

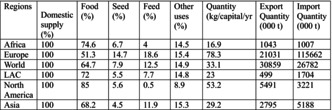

## 1 INTRODUCTION

The potato “(*Solanum tuberosum L*.)” is one of the most productive crops in the world. According to data from “the Food and Agricultural Organization (FAO)”, more than 300 million tons of potatoes were produced annually in 2016. Also, one of the most significant industries in the world, the food processing industry, creates a lot of organic waste that needs to be handled and managed improperly to prevent environmental damage and to spread further by promoting by‐product usage (Abebaw, 2020). The two primary categories of by‐products generated by the potato processing industry are wasted potatoes and processed potato wastes. The potato business has a disposal issue with both leftover tubers and potato by‐products since the moist wastes can cause plant rot and pathogenic illnesses (Joly et al., 2020; Rodríguez‐Martínez et al., 2021). The potato business is thought to produce between 12% and 20% of its overall volume in waste and by‐products. Peels, pulp, and rejections are waste products of the processing of potatoes. Peels, pulp, and unsalable potatoes can be further processed in starch plants, added to formulas for animal feed, or converted to ethanol. As a result, efforts to recycle industrial potato waste will give livestock more options for feed and increase the efficiency of potato production and processing. Nonetheless, despite persistent attempts, food waste is still an issue in our society. In some parts of the world, the majority of food loss occurs during harvest and storage, or it goes unconsumed by Western customers. Recent efforts to reduce potato waste include biotechnological techniques that use industrial waste as components of microbe growth media (Bzducha‐Wróbel et al., 2018). Such a strategy practically permits the manufacture of a new product with added value while also enabling the complete biodegradation of organic substances. Moreover, using waste products as intermediate components lowers overall production costs (Kot et al., 2020). To investigate and evaluate the degree to which industry potato wastes are responsible for adverse effects on the environment and living habitats, a thorough systematic study is conducted in this research. In addition, the biotechnological management and utilization methods of industry potato waste by‐products are described, together with their limitations and real‐world difficulties that function as roadblocks to their efficient application.

## 2 OVERVIEW OF COMMERCIAL USAGES OF POTATO

Potato farming is extremely important for many developing world populations, both economically and nutritionally. After rice (608 Mt) and wheat (630 Mt), it is the third most important crop that people consume in a quantity of over 300 million tons (Mt) annually. Because of its adaptability, production capacity, nutritional value, and role as an essential component of varied cropping systems, the food crop has a long history of lowering food insecurity and increasing household incomes throughout periods of crisis and current population expansion (Devaux et al., 2021). In terms of their fulfilling nutritional value, potatoes can grow in a variety of conditions and have a high food production value per unit area. They are one of the richest sources of antioxidants and have a great supply of protein, carbohydrates, vitamins, minerals, and dietary fiber (Wang et al., 2022). Niacin, vitamin B6, and vitamin C are all abundant in their tubers. Despite this, the potato plant has one of the highest input requirements for fertilizer, whose cost has been steadily rising over time. The two primary segments of the worldwide potato market are fresh and chilled potatoes. While chilled potatoes are shipped or imported and used in the food processing business, fresh potatoes are used for daily eating. Due to their long shelf life, potatoes have gained popularity even in nations that have not historically consumed them. In the processed food sector, where processed goods make up the majority of potato consumption, fresh potatoes are in extremely high demand globally (Fritsch et al., 2017). The top countries exporting fresh potatoes worldwide in 2020, “according to the International Trade Map, were France, China, Canada, the Netherlands, Germany, and the United States. In 2020, the largest potato‐producing nations worldwide were China, India, the United States, Russia, and Germany, with yields totaling 78.2 million metric tonnes, 51.3 million metric tonnes, 19.6 million metric tonnes, 18.8 million metric tonnes, and 11.7 million metric tonnes, respectively” (Mordor Intelligence, 2021). Due to the indigestibility of nongelatinized starch and the presence of antinutritional proteins in raw, unheated potatoes, potatoes intended for immediate consumption should be boiled before consumption. They are cooked by baking, boiling, or frying. In a variety of dishes, including mashed potatoes, potato salad, potato dumplings, fries/chips, potato soup, potato au gratin, potato pancakes, potato wedges, jacket potatoes, and hash browns/röstis. Various potato preparations can cause different nutrient losses, such as a 13% loss of ascorbic acid when cooking potatoes that have not been peeled versus a 41% loss when they have. It should be noted that potatoes have a higher risk of becoming obese, primarily due to their high glycemic index. Recent analyses of clinical intervention and observational research focused on the potato concluded that there was insufficient evidence to support any link between potato consumption and the hazards of obesity, type II diabetes, or cardiovascular disease (Devaux et al., 2021). Yet, the need for fried potatoes is growing as a result of the trend toward urbanization and associated lifestyles, rising earnings, and increased consumption of “convenience foods”. Overconsumption of these high‐energy foods combined with a lack of exercise can result in weight gain and obesity. To reduce obesity and diet‐related noncommunicable diseases including diabetes and heart disease, it is important to consider the function of fried potato products in the diet (Bzducha‐Wróbel et al., 2020).

Fresh potatoes are being replaced in terms of global potato consumption by added‐value processed meals including chips, fries, and dehydrated goods, as well as the manufacturing of starch and alcohol.

**FIGURE 1:**
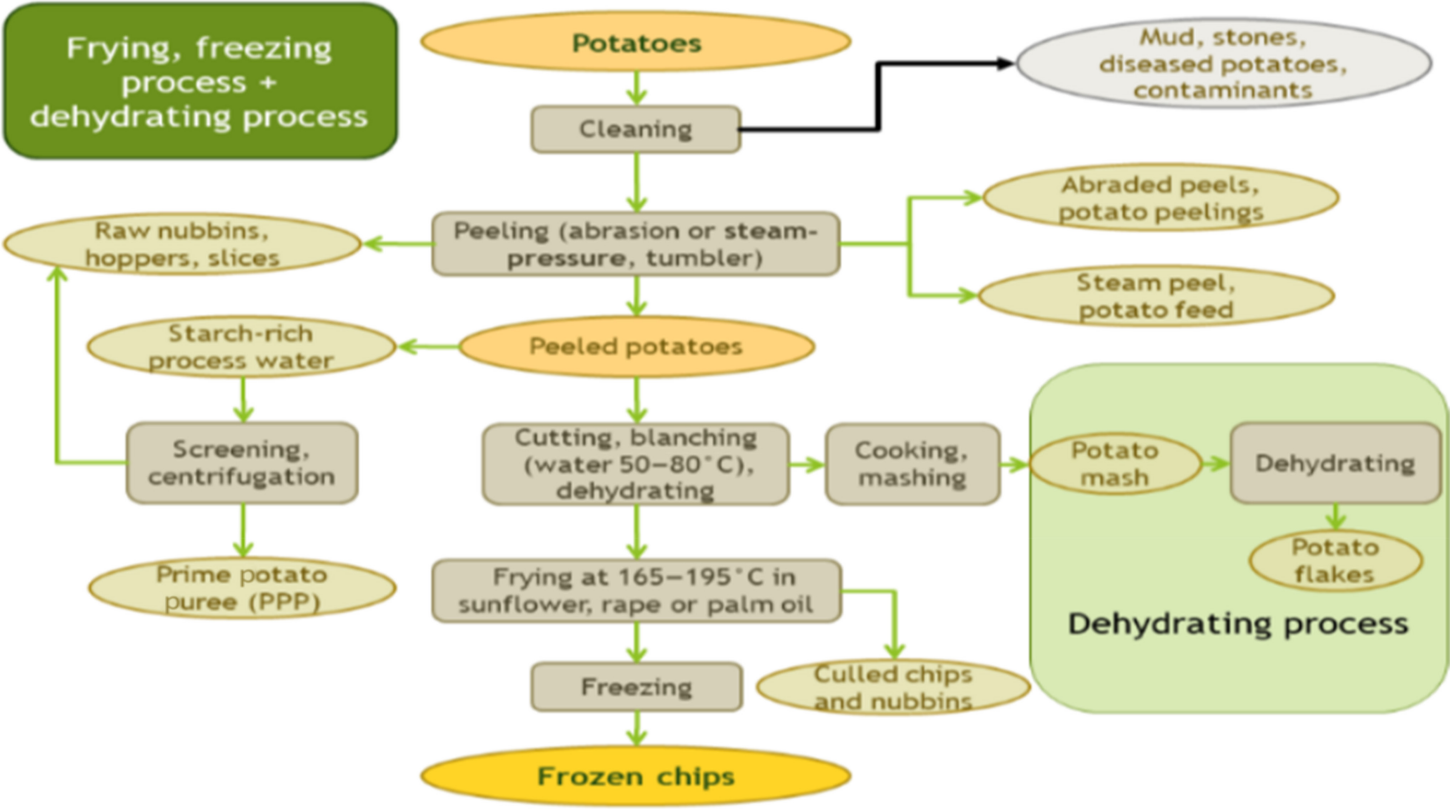
By‐products generation in industry potato processing methods. *Source*: Heuzé et al. (2020).

Between 50% and 60% of the potato crop is processed into food goods or other industrial products in North America and certain European nations. Between 2007 and 2017, the global commerce in frozen processed potato products expanded from 3 million tonnes to 7 million tonnes. Although the Netherlands, Belgium, the United States, and Canada are the top suppliers of these goods, Asia, the Middle East, and Latin America have seen the largest market growth (Table 1). Potato chips (crisps), fries (chips), and other frozen items are the main processed goods, followed by dried goods, chilled peeled potatoes, and tinned food (OECD, 2021; Figure 1).

The table below shows the global trend of potato utilization, consumption, and trading operation in recent dates.

**TABLE 1:** Global trend of potato utilization, consumption, and trading operations.

Regions	Domestic supply (%)	Food (%)	Seed (%)	Feed (%)	Other uses (%)	Quantity (kg/capita/year)	Export quantity (000 t)	Import quantity (000 t)
Africa	100	74.6	6.7	4	14.5	16.9	1043	1007
Europe	100	51.3	14.7	18.6	15.4	78.3	21,031	115,662
World	100	64.7	7.9	12.5	14.9	33.1	30,859	26,782
LAC	100	72	5.5	7.7	14.8	23	499	1704
North America	100	85	5.6	0.5	8.9	53.2	5491	3221
Asia	100	68.2	4.5	11.9	15.3	29.2	2795	5188

### 2.1 Commercial potato by‐products in agro‐food businesses

The diagram given below demonstrates the production of by‐products in various commercial potato processing methods, such as frying, freezing, and dehydrating.

According to Freeman (1996), Entrepreneur India (2021), and Haverkort et al. (2022), potatoes are often employed economically as agro‐food consumer products in the following ways: Potato tubers may be readily broken down and the starch and pulp separated to create the major component of potato dry matter, starch. Either whole tubers or processing wastes can be recovered to make starch. Uses—the primary raw material utilized to create both food and nonfood goods is starch. A large amount of starch is transformed into starch hydrolysate during food preparation.

Moreover, it thickens gravies and soups. In comparison with cereal starches, pregelatinized potato starch is utilized in large quantities in quick puddings because of its superior qualities. It is employed in the confectionery industry as a thickening agent in synthetic jellies, as a medium for molding cast candies like jelly beans and gum drops, and for sauces, soups, and stews.

When processing, potato starch has produced a significant amount of waste residue. Waste products from the potato starch industry's processing, such as effluents and potato leftovers, seriously impact the environment. A factory that produces 10 kt of potato starch annually releases 720 t of effluents and 192 t of leftover potatoes every day for 100 days. Potato residue is the thin, solid by‐product of starch extraction that has a water level of more than 80% and contains 33 isolates (28 bacteria, 4 fungi, and 1 yeast). It is difficult to store because of its high water content. According to the investigation, Chinese potato starch companies leak effluent and potato remains into waterways, seriously polluting them. The fresh waste residue contains a lot of water (nearly 80%), making it unsuitable for storage and long transport. Due to this, it is frequently dumped outside, where the organic materials can rot for many types of bacteria and pollute the environment by generating a foul odor. On the other hand, the price would be out of control if this potato waste residue were dried and turned into dry feed. However, even if it is immediately used as a feed or buried locally, it may pollute the soil and subsurface water. Potato powder is a healthy flour that is highly concentrated and made from boiled potato pulp. When breading meat and fish, potato powder is used as a thickening ingredient in soups and stems. The military, the civilian economy, and the school lunch program all make substantial use of it. The key difference between potato starch and potato powder is that potato powder is made by dehydrating fresh potatoes and includes the skin in addition to all of the other dry potato components. As potato starch is just one of many elements in potatoes, it lacks the nutrition, flavor, and taste of cooked potatoes. Potato powder after watering, on the other hand, maintains the integrity of potato cell granules as much as possible. In addition to having the same nutritional value as cereal flour, potato powder is also quite high in vitamins C and K. In the process of making potato chips, potato powders are produced on a huge scale as waste by‐products. Typically, during production, these wastes mix with the wastewater used to wash the chips. In addition, potato powders are a by‐product of potato peel wastes (PPWs) in a variety of industries that prepare potatoes for food. For the preparation of namkeen, bhujiya, soup curries, and snack foods, among other varieties of crispy food products, potato granules are utilized. It can be used to make food sweeter. To make potato granules and other food processing products, both organized and unorganized commercial sectors are involved. For smoother sauces, gravies, and soups, potato flakes or powder are added to bread, pancake, and waffle recipes and also utilized in the production of manufactured potato chips, extruded snacks, snack pellets, battered breaded items, etc. Cooking that is free of gluten and free from allergens also uses it. The usage of potato flakes or powder is expanding in several food preparations, including stir‐fries, soups, ready‐to‐eat vegetable curries, and snack foods like McD's, Pringle, and Haldiram Namkeens. It tastes strongly of potatoes. It can serve as a binding agent for making items like “kheer, tikki, chops, pakoda, cutlets, stuffed paratha, and kofta. Its use at present is mostly in hotels and restaurants, but adoption in the household is expanding due to its inclusion in things like ready‐to‐cook soups, dal, curries, etc.” It gives curries additional flavor. Waste potato mash is made from leftover potato flakes and contains 80% moisture on average. “The United States' Keystone Potato Products, LLC (Hegins, PA) produces potato product lines, such as potato flakes, with a potential of 7,700 kg/h, out of 900 kg of skin and 225 kg of low‐quality waste mash potato per hour (Personal communication with Keith Masser)”. As a direct consequence of these wastages, a significant amount of nutrients is lost (Izmirlioglu & Demirci, 2012). Otherwise, potato flakes or granules are formed as by‐product wastes following the heating of potatoes in the production of chips or of dehydrated puree, potato flour, and in the process of fake chips like “Pringles” reconstituted from potato puree. These wastes can be formed as liquids or semisolids, for example (Gebrechristos et al., 2020; Heuzé et al., 2020; Merino, 2021). As seen in potato waste processing, little potato chunks and flakes (0.55 cm in diameter), starch granules, and significant amounts of potato‐cell agglomerates are typically present in the wastewater after the separation of potato peels (PPs). The composition and total concentration of the solid matter component are highly variable (European Commission, 1999). In general, potato chips are consumed as snacks. Several potato types are typically used to make chips. Less sugar is present in potatoes used to make chips. Potato is used more frequently as a snack meal in the form of chips or wafers in addition to being a key ingredient in many veggie cuisines. One of the most popular ready‐to‐eat snacks, enjoyed in practically every country in the globe, is potato wafers and chips. They can be a snack, a side dish, or an entrée. To wash, peel, and blanch the raw ingredients for potato chips, a lot of water is required. These procedures produce liquid waste with a high starch concentration, often between 20% and 25% g/L.

### 2.2 Problems of potato waste contamination and food loss

The majority of research concludes that between 16% and 25% of the initial weight of potatoes is wasted during processing. “The anticipated total world production for potatoes in 2020 was 359,071,403 metric tonnes”. This results in a waste stream that is predicted to reach at least 44 million metric tonnes globally if 60% of all potatoes are processed (Hong et al., 2020; SavFood, 2022).

From whole potatoes to tiny fragments, raw materials that cannot be processed come in a variety of sizes. They contribute greatly to food loss and contamination. Raw pulp, as used in this context, is finely separated pulp from raw potatoes. Cutting scraps, peeler waste, and pulp from starch separation are examples of sources that yield raw pulp. Finely, separated raw potato particles will be released when cleaning equipment is used to handle raw potatoes. Fine screening or settling are two methods for removing the raw pulp from the waste stream (Gebrechristos & Chen, 2018). The potato tuber's intercellular bonds are broken by the softening effect of heat during peeling or processing procedures, which causes considerable cell separation and clumps of cells to form during the washing and handling phases. They swiftly scatter in the effluent. The majority of these agglomerates pass the typical 20‐mesh screen aperture; however, screening removes many of them. These solids are a significant component of the settleable solids removed during the primary treatment of waste streams from the processing of potatoes. They settle quickly in a clarifier that is appropriately configured. The readily water‐soluble potato components show up as dissolved particles in the final waste stream. They consist of carbohydrates, proteins, amino acids, and solubilized starch. The only way to get rid of this organic waste stream component is secondary treatment, more precisely some kind of biological oxidation or land disposal. The majority of the wastes generated by the potato processing industry are useless organic materials that, when disposed of on land, pollute the environment because they are easily attacked by microbes. Leaching problems also occur when wastes with a high moisture content are disposed of in public sanitary landfills (Jacob et al., 2016). The main issues with potato waste include its simple decomposition, which causes acidification and quickly decreases the pH considerably. This issue is linked to the substrate's low pH, poor buffering ability, and potential for significant volatile fatty acid (VFA) formation during digestion. The starting phase of anaerobic digestion is the most important stage, because failure to do so results in process disruption and instability. In the worst‐case scenario, VFA buildup drastically inhibits methanogenesis. Particularly vulnerable to the effects of unchecked acidification are single‐phase systems. Generally speaking, methanogens are obligate anaerobes that are sensitive to low pH. (B5). Cull or waste potatoes, on the other hand, are any tubers that are deemed unsuitable for the fresh, processing, or seed potato markets. Infection (both in the field and storage), bruising, unfavorable weather conditions, undesirable size, and a lack of markets all lead to culling potatoes. These potatoes must finally be disposed of. The fungus known as late blight (*Phytophthora infestans*) can make culls a more significant issue. The spread of late blight throughout a field and from one crop to another can be incredibly fast and possibly quite devastating. Spores produced by late blight can be transmitted by wind, water, machinery, or people. In the spring, the scent from a cull pile might become an annoyance. If the pile is next to a residential area, the smell produced by rotting, damp tubers can be problematic. The decaying pile also draws insects, like the Colorado potato beetle. The beetles will consume the vegetation in the pile before moving on to nearby or neighboring fields. Problems with aphids can also arise. They might drink the plant juices off the mound and then travel to a neighboring field. If the pile contained either the potato mosaic virus or the potato leaf roll virus, there is a potential for disease transmission. Cull piles next to waterways are especially problematic because nitrates produced from tuber decay could contaminate adjacent water sources (Saskatchewan Agriculture, 2020). Because potato processing wastewater contains high concentrations of biodegradable components such as starch and proteins, “in addition to high concentrations of chemical oxygen demand (COD), total suspended solids (TSS) and total Kjeldahl nitrogen, the potato processing industry presents potentially serious water pollution problems. An average‐sized potato processing facility producing French fries and dehydrated potatoes can create a waste load similar to that of a city of 200,000 people. Approximately 230 million liters of water are required to prepare 13,600 tonnes of potatoes”. For every kilogram of potatoes produced, this equates to around 17 L of trash. Wastewater from the processing of raw potatoes can have up to 10,000 mg/L of COD. Also possible are values of 9700 mg/L for TSS and 9500 mg/L for volatile suspended solids. The components of the waste stream that results from potato processing processes depend on the type of potatoes utilized. Dirt, caustic, fat, cleaning, and preservative chemicals are a few of the foreign substances that could be present in the potato. Typically, several waste streams are mixed to form effluent before being discharged from the potato processing plant. Because different processes produce different amounts of wastewater using different processing techniques, it is challenging to generalize these amounts. Many research and references in this regard demonstrate the wide variances in water usage, peeling losses, and waste flow reporting techniques (Hung et al., 2006).

## 3 BIOTECHNOLOGICAL INDUSTRY POTATO WASTE MANAGEMENT AND UTILIZATION METHODS

The utilization of local resources and waste in the effective creation of value‐added goods is a technique known as the “bio‐economy.” The world recognizes biotechnology as the current trend in industrial processes, as opposed to the conventional way of product synthesis via chemical synthesis. The argument is that whereas the latter method contributes to waste and environmental contamination, biotechnology processes are advantageous for the environment. Many issues with traditional techniques of pollutant treatment through landfills or incineration have fueled the demand for innovative, cost‐effective, and dependable biological ways of pollution treatment (Ezeonu et al., 2012). Using techniques for converting food waste into value‐added commodities, the environmental issue caused by food waste is resolved, and economically feasible manufacturing methods are offered. Food waste comes in a variety of forms, including liquid, solid, and semisolid wastes such as wastewater, fats, spent cooking oil, dangerous household items, and others. It is well known that these wastes could have harmful effects on the environment and human health. Water use in significant quantities for activities such as cleaning, sanitation, cooking, and transportation results in the production of liquid waste. Furthermore, lignin, cellulose, amylose, and monosaccharides are used to compress solid wastes, expressing nutrients in a polluted form. Even though the price of fossil fuels, the depletion of natural resources, and the rising costs of natural resources are inevitable, the main factors driving the expansion of environmentally friendly technologies based on less expensive food products to achieve global goals of biofuels, chemicals, and biomaterials are their rapidly growing vitality (Sufficiency et al., 2022). Moving on to the primary topic of this article, peeled and damaged potatoes account for the majority of potato production waste. When bio‐economy principles are taken into account, it is possible to utilize this waste in pharmacy, food production, and therapeutic purposes. Although it is mostly used for the manufacture of animal feed or biofuels, it contains significant amounts of useful components. The treatment and use of potato wastewater for nutrient recovery and the creation of other sustainable resources is also an issue. This section presents and discusses the most recent advancements in the application of biotechnological technologies for the management and use of potato wastes with added value.

### 3.1 Biotechnological waste management and utilization of potato starch

Granules of potato starch are typically B‐type crystalline and range in size from 25 to 100 m. Moreover, potato starch would develop thermoplastic properties when exposed to plasticizers, high temperatures, and shearing conditions, making it appropriate for creating a biodegradable film. Because of their weak water resistance, potato starch‐based films still need to perform better to expand their use in food packaging. To solve this problem, plasticizing agents and other active components were added to give films several benefits. The most starch‐compatible plasticizing agent that can provide polymers flexibility and robustness is reportedly glycerol. Moreover, gelatin, an animal‐derived protein, demonstrates high thermos reversibility and heat stability, offering potential barrier performance (Niu et al., 2021; Rasheed et al., 2023). The innovative ultrafiltration technology was used to get the technological and antioxidant characteristics of potato juice (PJ) protein concentrate. Functional qualities such as the ability to absorb water or oil, the ability to foam, the durability of the foam, and the solubility at various pH levels were evaluated. Moreover, antioxidant activity, mineral composition, and the total amount of phenolic compounds were measured. The outcomes demonstrated that the ultra‐filtered PJ protein concentrate has good oil absorption properties, which are more than twice as good as the commercial proteins utilized for comparison. Furthermore, improved was the capacity to produce and maintain the foam. In comparison with other samples, it had a higher level of macro‐ and microelements as well as antioxidant activity (Jeżowski et al., 2020). Two methods—direct acid hydrolysis and enzyme pretreatment followed by acid hydrolysis—were used to create starch nanocrystals. In 12 days, direct hydrolysis reduced the starch granules to nanocrystals. By pretreating the enzymes with amylase and amyloglucosidase, two starch‐hydrolyzing enzymes, the time needed to create starch nanocrystals was reduced in half. Both treatments produced starch nanocrystals of the ideal size, ranging in size from 10 to 50 nm (Raigond et al., 2018). The creation of drinkable alcohol from food waste is a remarkable option. The worldwide spirits manufacturing industry is expanding due to rising premium brand demand and rising per capita spirit consumption. Generally speaking, the process of producing ethanol from starchy biomass includes several processes such as liquefaction, saccharification, and fermentation. Chintagunta et al. (2016) used cocultures of “*Aspergillus niger* and *Saccharomyces cerevisiae*” to increase the generation of ethanol by simultaneous saccharification and fermentation in their waste utilization experiment with potato starch. *A. niger* and *S. cerevisiae*'s synergistic metabolic interactions in a starch medium increase amylolytic activity and total ethanol output by preventing the buildup of inhibitory concentrations of reducing sugar.

After being further improved, the nutrients found in the solid waste left over after the manufacturing of ethanol can be utilized to create bio‐manure, a useful way to lessen the amount of solid waste that accumulates in the environment. With humus improving soil structure and all the nutrients required for plant growth, the enhanced residue is good organic manure (Chintagunta et al., 2016).

### 3.2 Biotechnological waste management and utilization of potato peels

Although being one of the most common foods consumed by humanity, between 50% and 60% of the raw material used to produce potatoes is waste. Peels make up the majority of the piece. Typically, peeling occurs during the processing of potatoes, and depending on the method used, production losses in the form of PPW can range from 15% to 40%. Each year, following industrial potato processing, enormous amounts of PPW are left over. Although it has not been done yet, using PPs to make items with added value is a possibility. PPs may be used to make biodiesel, bio‐manure, biogas (methane), lactic acid, glycoalkaloids, phenols, and bio‐sorbent, which is a substance that removes pollutants from water. The peel chosen for manufacture depends on its composition (Priedniece et al., 2017).

The value‐added products made from the fermentation of PPs can be used in a variety of ways, including as adsorbents, bio‐composites, and packaging materials. They can also be used as dietary fiber or medical treatments. These items can be utilized to provide energy, create biopolymer films, limit corrosion, and create cellulose nanocrystals. The bio‐refinery strategy for PP will raise the value of this waste by creating a variety of products with value additions and lowering significant waste creation.

PPW can be converted into products such as biofuels, dietary fiber, biofertilizers, biogas, biosorbent, antioxidants, and food additives through various processes like fermentation, extraction, and other treatments. The application of PP utilization in food and nonfood reasons, such as “extraction, utilization of bioactive components, biotechnological usage, livestock feed, and other uses, was clarified by Javed et al. (2019)”.

Under controlled solid‐state growing conditions, PP is an incredibly affordable and efficient substrate for the production of thermo‐stable nascence‐amylase, a bounce hydrolyzing enzyme that is extensively used in colorful food products. Moreover, PP has been utilized in previous experiments as a low‐cost medium to manufacture the enzymes nascence amylase and alkaline protease, both of which have implied uses in cleansers (Abebaw, 2020). To produce bioplastics from industrial food waste that is not of food quality, designers Rowan Minkley and Rob Nicoll of London established Chip[s] Board. McCain, a family‐run company well‐known globally for its potato‐based products, provides the PPs used to make the product. Strong, recyclable, biodegradable, and chemical‐free materials are made from food waste (Chip[S] Board, 2022). It has been said before that there is a sizable market for bio‐ethanol made from potato trash. The mandated blending of bio‐ethanol with regular gasoline in levels up to 10% will result in a demand for enormous amounts of bio‐ethanol, should federal government regulations be approved in light of the Kyoto Protocol. The amount of starch, cellulose, hemicellulose, and fermentable sugars in PPW is sufficient to serve as a feedstock for ethanol synthesis. Driven by the demand, Arapoglou et al. (2010) tested the fermentability and ethanol generation of many batches of PPW that had been hydrolyzed with various enzymes and/or acids and fermented by “*Saccharomyces cerevisiae var. bayanus*.” The findings show that PPW, a potato industry by‐product, has a significant potential for ethanol generation.

### 3.3 Biotechnological waste management and utilization of potato pulp wastes

Because it contains a lot of carbohydrates, potato pulp waste (PPW) from a factory that processes potatoes needs to be degraded before it can be released into the environment. Testing of single‐chamber microbial fuel cells' ability to produce power from these wastes has been conducted (MFCs). The interactions between exoelectrogens and bacteria that break down polysaccharides in the anode biofilms, known as syntrophic interactions, were what caused the cascade utilization of PPW in MFCs (Tian et al., 2017). A promising area of application for potato pulp is the use of mixed techniques, which combine fermentation and physicochemical processes. “*Bacillus licheniformis*, *Aspergillus niger*, *Trichoderma asperellum*” for protein extraction, citric acid for pectin extraction, “*Acremonium cellulolyticus*” for saccharification of starch residues during ethanol production, which when used before or during fermentation yields a sizable amount of physiologically active compounds. Such advancements could boost the market for nutritious additives based on potatoes and healthy foods (Rubanka et al., 2021). Once more, pulp is a preferable raw material (fresh, dried, extruded aggregate) for the manufacturing of other foodstuffs and can fully or partially replace potatoes in the production of snacks, flour in the creation of long cookies, soybeans in the production of meat products, and so forth. However, such technologies require greater investigation and analysis. “In the current work, a unique strategy to combat the acidity issue has been tried by co‐digestion industrial potato waste (PW) with Pistia stratiotes (PS, an aquatic weed). With a substrate concentration of 5 g total solid (TS)/L (2.5 g PW + 2.5 g PS), the performance of co‐digestion of the weed and PW was investigated, and the results showed an increase in methane yield of 76.45% as compared to mono digestion of PW with a beneficial synergistic effect. Artificial neural network (ANN) coupled genetic algorithm (GA) model and central composite design (CCD) based response surface methodology (RSM) were used to optimize process parameters” (Jacob et al., 2016).

### 3.4 Biotechnological waste management and utilization of potato chip wastes

Goyzueta‐Mamani et al. (2020) evaluated the possible use of by‐products from the potato chip industry as a carbon source to develop a cost‐effective growing medium for *Mortierella alpina* to produce biomass, lipids, and arachidonic acid (ARA). The potential use of potato chip industry wastes as carbon sources for *M. alpina*'s production of biomass, lipids, and ARA was evaluated and characterized using a synthetic growth medium that was optimized using a Plackett–Burman and central composite rotatable design. This study showed that, in comparison with the traditional synthetic culture medium, using waste from the potato chip industry as a substitute source of carbon and macro/microelements, supplemented with a cheap yeast extract substitute, resulted in a 7% reduction in culture media costs when *M. alpina* biomass was produced with ARA enriched. To produce biomass, lipids, and ARA, it is proved that potato chip wastes can be used as a cheap source of carbon. This method is an environmentally friendly alternative to using agri‐food wastes to produce significant metabolites.

### 3.5 Biotechnological waste management and utilization of potato powder wastes

The only substrate for the concurrent generation of antifungals and biopigments was prepared and supplied to *Streptomyces* spp. The capacity of strain SO6 to produce intracellular biopigments and antifungals against commercially important fungal phytopathogens using powdered potato waste without the addition of additional nutrients made it stand out among the three different *Streptomyces* isolates. This strain also shows the capacity to release several enzymes for the fermentation of eight sugars that may be involved in the bioconversion of potato trash (Schalchli et al., 2021). The only substrate for the concurrent generation of antifungals and biopigments was prepared and supplied to *Streptomyces* spp. Out of three separate *Streptomyces* isolates, strain SO6 stood out due to its capacity to produce intracellular biopigments and antifungals against commercially important fungal phytopathogens using powdered potato waste without the addition of additional nutrients. Also, this strain showed the capacity to release a range of enzymes for the fermentation of eight sugars that may be involved in the bioconversion of potato waste (Schalchli et al., 2021). Making pigment from *M. purpureus* Went NRRL 1992 using potato waste powder has some advantages.

### 3.6 Biotechnological waste management and utilization of potato wastewater

Glycerol and deproteinized potato wastewater are two by‐products that are challenging to get rid of. It has been discovered that *Rhodotorula glutinis* obtained its carbon and nitrogen from glycerol and potato wastewater, respectively. The examined strain was grown on a medium containing 5% glycerol, and this resulted in the greatest degree of glycerol content reduction (70.6%). In this medium, it was found that the total protein concentration had significantly decreased, by an estimated 61% (Kot et al., 2020). An industrial wastewater treatment facility in Pennsylvania that was overrun with new potato waste used Probiotic Solutions Bio Energizer for 2 months. The results included increased microbial activity, decreased filament and foam, improved decanting, and decreased accumulated sludge and sludge hauling costs. Before, when fresh potato waste stream flows grew by 26% to 180,000 gallons per day, the plant was having issues with process control (GPD). In the sequencing batch reactor (SBR), the additional load was resulting in filamentous issues, settlability challenges, and higher sludge trucking expenses (Probiotic, 2019; Wei et al., 2022). Industrial trash is expensive and difficult to recycle, whereas organic waste from plants and animals, such as potato wastewater, whey, lignin, and cellulose, predominates and enumerates the potential applications of microorganisms in the use of different waste items. Using yeast biomass can help save money on waste disposal expenses. Using yeast biomass for waste management, it is now feasible to produce new metabolites with a variety of industrial uses, including β‐glucans, vitamins, carotenoids, and enzymes (Kieliszek et al., 2020).

A field experiment was conducted in Northern Italy (Po Valley), within the context of the EU project SAFIR, to analyze the impact of treated wastewater reuse on potato yield, quality, and cleanliness. *Escherichia coli*, a fecal indicator bacterium, and the presence of heavy metals in treated wastewater, soil, and tubers were examined.

Both tap water and recycled water produced roughly the same amounts of potatoes overall, but the latter produced more than was commercially viable. Reused water did not affect the tubers' sugar levels or dry matter content. With MBR and FTS water, the total sugar content was greater. Reused water greatly improved water use efficiency. With FTS and MBR, respectively, crop gross margin rose by 635 and 765 euros per hectare per year as compared to tap water (Battilani et al., 2014).

Torres et al. (2020) conducted research on the integral valorization of discarded potatoes from three local types utilizing low‐impact procedures to recover the starch found in the flesh as well as the bioactive chemicals found in the skin or the processing wastewater. Following starch extraction, the residual flesh was also collected for processing (Table 2).

Potential uses for both food and nonfood items have been suggested as a result of the extraction of starch and active extracts using environmentally friendly methods, their physicochemical and phytochemical characterization, and the formulation and mechanical characterization of the corresponding functional hydrogels. According to the findings, subcritical water extraction is a reliable approach for reclaiming antioxidants from potato skin. High levels of protein were found in processing wastewater. The extracted starch had physicochemical qualities that were comparable to those of commercially available starch, and the related hydrogels showed improved mechanical properties without syneresis.

A summary of the above‐discussed review is provided in the table below.

**TABLE 2:** Description of the biotechnological application of potato waste.

References	Potato by‐product waste studied	Biotechnological tool used	Significance
Niu et al. (2021)	Potato starch	Exposed to plasticizing agents, such as glycerol and gelatin, an animal‐derived protein	Thermoplastic properties enhanced with polymer flexibility and robustness
Jeżowski et al. (2020)		Innovative ultrafiltration technology	Antioxidant characteristics; absorption of water or oil; ability to foam; durability of the foam; solubility at various pH levels
		Direct acid hydrolysis and enzyme pretreatment	Starch nanocrystals formation
Chintagunta et al. (2016)		Cocultures of *Aspergillus niger* and *Saccharomyces cerevisiae*	Bioethanol formation
Priedniece et al. (2017)	Potato peels	Based on multiple reviews on biotechnological Tools applicable to potato peel wastes	Formation of biodiesel, biomanure, biogas (methane), lactic acid, glycoalkaloids, phenols, and biosorbent
Pathak et al. (2017)		Fermentation	Formation of adsorbents, biocomposites, packaging materials, dietary fiber, or medical treatments
Javed et al. (2019)		Fermentation, extraction, and other treatments	Bioactive components, biotechnological usage, livestock feed
Abebaw (2020)		Enzyme extraction	Resource of colorful food and cleansers
Chip[S] Board (2022)		Biofilm	Bioplastics formation
Arapoglou et al. (2010)		Hydrolysis with various enzymes and/or acids and fermentation by *Saccharomyces cerevisiae* var. bayanus	Bioethanol formation
Tian et al. (2017)	Potato pulp	Syntrophic interactions between polysaccharide‐degrading bacteria and exoelectrogens	Microbial fuel cell (MFC)
Rubanka et al. (2021)		Fermentation and Physicochemical processes by using “*Bacillus licheniformis*, *Aspergillus niger*, *Trichoderma asperellum* (for protein extraction), citric acid (for pectin extraction), *Acremonium cellulolyticus* (for saccharification of starch residues during ethanol production)”	Formation of nutritional additives
Jacob et al. (2016)		Codigestion industrial potato waste (PW) with Pistia stratiotes (PS, an aquatic weed)	Replacement of potatoes in snacks and foodstuff with low acidity risk
Goyzueta‐Mamani et al. (2020)	Potato chips	*Mortierella Alpina* growth	Biomass, lipids, and arachidonic acid (ARA)
Schalchli et al. (2021)	Potato powder	*Streptomyces* isolates, Strain SO6 that can also be used for enzyme extraction	Production of antifungals and biopigments
Kot et al. (2020)	Potato wastewater	*Rhodotorula glutinis* growth	Glycerol and protein content reduction
Kieliszek et al. (2020)		Yeast biomass growth	Extraction metabolites such as β‐glucans, vitamins, carotenoids, and enzymes from potato wastes; Waste treatment cost reduction
Battilani et al. (2014)		*Escherichia coli* growth	Comparing the treatment in wastewater and tap water treatment, commercially feasible reusable potato waste yield was found more in wastewater
Torres et al. (2020)		Physicochemical and phytochemical analysis; formulation and mechanical characterization of the corresponding functional hydrogels	Starch and bioactive component recovery from potato wastes

### 3.7 Challenges in biotechnological industry potato waste management and utilization processes

Diverse and large‐scale production of industry potato by‐products poses a serious challenge for commercial potato product industries leading them to rethink sustainable and cost‐effective potato waste management strategies. Biotechnological processes that are currently applied as part of sustainable potato waste management options lack in terms of their potential efficacy of using suitable biodegradation schemes of potato waste utilization.

This is because there are still few convincing arguments supporting the viability and efficacy of biotechnology‐based instruments, such as the use of biosensors, biorefineries, or the selection of appropriate biocatalysts for techniques of biodegrading or converting potato waste. The issue for biotechnology is to give the concept of industrial sustainability the push and the right tools for widespread adoption.

Piecemeal, short‐term solutions are no longer appropriate for handling environmental issues. “The difficulty with this fractured approach is that it addresses a race of new issues without inescapably resolving the former one (s), thereby creating the print that (they) no longer counts. Attention focuses on one subject, overshadowing others that are no less important. This approach also fails to treat the terrain as a single system, which makes it nearly insolvable to show people how they affect the terrain” (Dutch Ministry of Housing and Environment, quoted by OECD, 1998).

Very large volumes of raw materials will be required to develop the biotechnology industry on a wide scale, and the sources will need to be diverse to implement effective biotechnology‐based industry potato waste management and utilization system. The primary sources of carbon used in the biotechnology industry's basic materials are starch, sugars, and in the coming years possibly cellulose.

The problem is especially important because agricultural activity produces these carbon‐containing chemicals. Situated at the intersection of agricultural and nonfood businesses, it will not always be simple to use enough potato waste components of the appropriate grade.

Once more, the main barrier to the commercial use of soluble enzymes for environmental applications is their low operational stability, which necessitates the need for an ongoing supply of significant amounts of enzymes. Immobilizing the enzyme increases its half‐life and operational stability, which lowers the treatment cost. The purified enzyme, pricey supports, and expensive reagents are frequently needed in enzyme immobilization techniques. This process enables the reuse of enzymes for numerous reaction cycles. This means, therefore, that the preparation of immobilized enzymes is more expensive (Lončar & Vujčić, 2011).

Patents are another issue in biotechnology. Determining the patentability of items or processes that are based on living things is more challenging than doing so for more traditional technological ideas. The utilization of biocatalysts, genetic engineering, and microorganisms are three areas where patenting issues frequently occur. The majority of biocatalysts for treating phenolic wastewater are still not commercially available, as shown in potato wastewater treatment plants. The adaptation of large‐scale enzymatic treatment of industrial wastewater could, therefore, provide a significant economic and technical barrier (Salehi et al., 2021).

The experts also want to emphasize the importance of safety. The issue extends beyond genetic engineering and microbiology; it specifically affects industrial waste utilization systems that deal with massive amounts of microorganisms. Several of the most prominent scientists are confident that, above a certain scale, it will be almost difficult to ensure that there would be no microorganism leakages during production itself or the crucial stages of molecular purification and recovery (Bull et al., 1982).

## 4 THE USE OF PJ WATER AS A MICROBIAL MEDIUM FOR THE PRODUCTION OF METABOLITES

The food sector produces a large amount of wastewater that is high in biogenic components and organic compounds. Their applications are time consuming and expensive. According to Kieliszek et al. (2020), some potato processing waste, such as wastewater created during starch synthesis, can be used to provide yeast with nitrogen and mineral components. It is estimated that processing 1000 Mg (megagrams) of potatoes yields 600 m3 of potato effluent (Bzducha‐Wróbel et al., 2015). New methods of utilizing wastes had to be developed as a result of restrictions the European Union placed on the release of industrial effluent into the environment. One of them is to employ cheap microbiological medium made of liquid waste from the food sector. Potato wastewater is a significant source of vitamins and proteins, mostly the B group, and contains both inorganic and organic components (1% and 4%, respectively; Kot et al., 2020; Kowalczewski et al., 2019).

The efforts of Abdelraof et al. to use PPW as a source of substrates for bacterial cellulose (BC) biosynthetic media were recently documented. However, acidic hydrolysis with additional chemicals, considerable energy consumption, and additional reagents were also necessary when processing PPs to acquire appropriate sugar content. This method has little industrial application and is not very cost‐effective (Abdelraof et al., 2019).

In spite of the absence of any pretreatment, the BC yield from the PJ medium (>4 g/L) was comparable. The macro‐ and microstructure, and chemical composition of pPJ and control BC did not differ significantly either. Importantly, the BC isolated from PJ was not cytotoxic to the fibroblast cell line L929 in vitro and did not contain any difficult‐to‐remove contaminants. Similar to BC obtained in the standard medium and supplemented with antiseptic, the PJ‐BC soaked in antiseptic had an antibacterial action on *Pseudomonas aeruginosa* and *Staphylococcus aureus*. From an application perspective, especially in biology, these are very significant factors. Therefore, using PJ for BC biosynthesis is a path toward significantly increasing the value of a waste product of the starch industry that is environmentally problematic as well as toward significantly lowering the cost of producing BC, allowing for wider use of this biopolymer in biomedicine (Ciecholewska‐Juśko et al., 2021).

### 4.1 Scope and future possibilities of biotechnological industry potato waste management and utilization

Despite the limitations of feasible methods, lack of availabilities of suitable biotechnological tools, and unoptimized policy measures of removing/utilizing industry potato wastes, they are used and scientifically explored particularly for their eco‐friendly aspects as well as economic benefits. Following are the areas that need more attention to enhance the application efficacies of biotechnology in:

- Analysis of the availability and efficacy of biocatalysts (enzymes, microbial activities, life phases, etc.) that are effective in minimizing industry potato wastes or making eco‐friendly reuse.
- Suitable inclusion of biotechnological tools in wastewater treatment plants that operate industry potato wastes to recognize microbe–potato waste interactions and their risks or prospects.
- Efficacy measure of biotechnological tools to extract bioactive components from potato wastes or alter its physiochemical traits to help in utilizing in making eco‐friendly products, such as biomass, ethanol, bioplastics, biofuel, etc.
- Analysis of contamination of potato wastes and their interactions with other harmful contaminants (heavy metals, organic contaminants, dissolved gaseous matters, etc.) and the role of biotechnological tools to minimize their severity.

## 5 CONCLUSION

The use of biotechnological tools in minimizing industry potato waste is being emphasized in recent times on account of their sustainability, economic and eco‐friendly aspects. Nevertheless, these methods need more evaluation and enhancements to achieve optimality when they are used for organic waste reduction or their utilization, they are already proven useful to implement sustainable, eco‐friendly, and economic tools of organic waste management and utilization. The limiting areas, such as identification of the right biocatalysts, adequate policy implementations in wastewater treatment plants for potato waste reduction, and proper biotechnological control tools to minimize harmful interactions of potato wastes with other aquatic contaminants should be extensively analyzed and evolved for betterment. Further, a combined system of biotechnological tools, that is multiple biocatalysts, biosensors, filters, and others can be employed in building up effective biorefinery to identify and analyze the potato waste attributes, their removal need, and utilization potency. PJ‐BC should, therefore, be usable in the same applications as BC made commercially. In addition, the vast availability and low cost of PJ, a by‐product of the potato starch industry, should make it reasonably simple for achieving the conversion of the BC process in order to employ PJ medium at an industrial scale.

## AUTHOR CONTRIBUTIONS

**Anamika Chauhan:** Writing – original draft (equal). **Fakhar Islam:** Writing – review and editing (equal). **Ali Imran:** Validation (equal). **Ali ikram:** Software (equal). **Tahir Zahoor:** Formal analysis (equal). **Sadaf Khurshid:** Resources (equal). **Mohd Asif Shah:** Visualization (equal).

## CONFLICT OF INTEREST STATEMENT

The authors declare that they have no conflict of interest.

## ETHICS STATEMENT

The study does not involve any human or animal testing.

## Data Availability

Additional data are available on request from the corresponding authors.
